# Determination of Bone Density by DEXA Method Based on Bone Age and its Comparison with Chronological Age in Chronic Patients

**DOI:** 10.31138/mjr.34.1.44

**Published:** 2023-02-21

**Authors:** Leyla Katebi, Ali Rabbani, Fatemeh Sayarifard, Mehrzad Mehdizadeh, Azadeh Sayarifard, Arya Sotoudeh, Farzaneh Abbasi, Parastoo Rostami

**Affiliations:** 1 Department of Pediatrics, Faculty of Medicine, Ardabil University of Medical Science, Ardabil, Iran,; 2 Department of Pediatrics, School of Medicine, Children’s Medical Center, Tehran University of Medical Sciences, Tehran, Iran,; 3 Department of Radiology, Faculty of Medicine, Tehran University of Medical Science, Tehran, Iran,; 4 Department of Community Medicine, Faculty of Medicine, Tehran University of Medical Science, Tehran, Iran

**Keywords:** paediatric, chronic disease, bone densitometry, DEXA

## Abstract

**Background and Objective:**

Given the growing awareness about the important role of children’s age in building bone for a person’s life, physicians need to assess bone health in high-risk children for bone density disorders more than before to optimize their bones’ density and prevent osteoporosis in future. The aim of this study was to evaluate bone density based on chronological and bone age.

**Materials and Methods:**

In this cross-sectional study, 80 Patients who have been referred for bone density to the Osteoporosis Centre of the Children’s Medical Centre over a one-year period (spring 98 to spring 99) were studied. Bone density was performed for all patients by using DEXA method.

**Results:**

The z-score mean chronological age for the lumbar spine was −0.8± 1.85 years and bone age was −0.58±1.64 years. The z-score mean chronological age for femoral bone was −1.6±1.02 years and bone age was −1.32± 1.4 years.

**Conclusion:**

Results showed that in all patients, the difference in the mean Z score of chronological age and bone age of the spine between patients was not significant but for femur was significant. Also, use of corticosteroids leads to significant difference between the two age groups’ z-score in femur and spine.

## INTRODUCTION

Given the growing awareness of the important role of infant age in formation of bone for life, physicians consider it more important than ever to assess bone health in children who are at risk of bone density disorders or even in healthy ones, in order to help their bones to be optimized and prevent osteoporosis by increasing density.^1^ Awareness of the patient’s growth potential is one of the important points to be successful in various treatments. So, some indicators such as age, sexual maturity, skeletal growth stages, dental development stages, height, and weight are used to evaluate the growth stages. One of the most important growth indicators of people is their bone density.^2^ Although the diagnosis of osteoporosis is not based solely on measuring bone mineral density during growth, it can be helpful in following the patients with primary and secondary osteoporosis.^1^ According to the World Health Organization (WHO), osteoporosis is defined as a decrease in bone density by 2.5 points of the standard deviation from the mean maximum bone density in young and normal individuals of the community (T-score <−2.5). The decreased bone density between −1 to −2.5 points, less than mean maximum bone density in young and normal individuals of the community is called osteopenia, and the higher points would be considered normal (T-score < −1). It is noteworthy that osteoporosis in men under 50 years of age and premenopausal women and children is diagnosed based on the presence of fracture fragility or low Z-Score along with other risk factors of fracture. In cases that the Z-Score is less than −2, the term “low bone density” is used, and if it is greater than −2, the term “within the expected range” is used.^3^ Another indicator of growth is a person’s age, which may be different ages in a person’s body, such as chronological age, height age, bone age, and so on. There are significant differences in children’ development with the same chronological age, which indicates the need to understand physiological or biological age. Physiological age is the rate of progress toward puberty that can be estimated by dental, skeletal, sexual, or physical maturity.^4^

The classical method of bone age assessment is based on recognizing the apparent changes in radiography and maturity indicators in left hand radiography, which is measured in comparison with a reference atlas.^5^ The bone age can be used as a diagnostic or following method in patients suffering from growth or puberty disorders, and in some cases as a prognosis predictor for a person’s final height. A child’s bone age indicates the stage of biological and structural maturity better than the chronological age. Hand and wrist radiography is the most common method for calculating bone age. Automated methods for evaluating hand and wrist radiography have also been developed that reduce the technician based effects (on the study) compared to the manual method.^6^ Research has shown that there is a significant correlation among chronological age, puberty and skeletal age.^7^ In some cases, specifically chronic diseases and diseases need for steroid therapies, such as cystic fibrosis, Juvenile rheumatoid arthritis, and thalassemia, the development of the growth and height might be affected by the disease and therapies.^8^

International guidelines recommend assessing the bone density in children with particular chronic diseases.

Given that most of these children suffering from growth disorders, short stature and disproportionated bone age to the chronological age, so it is difficult to evaluate the bone density status. The DEXA method is currently used to assess bone density. DEXA can be used to estimate the exact structure of the body with minimal patient cooperation. Accurate interpretation of the obtained data by DEXA in children requires attention to bone size, stage of maturity, skeletal maturity, race, and body composition.^9^ Low bone density in children according to the Z-score criteria is defined as less than −2SD. In screening, bone mineral density (BMD) of the spinal cord is more valuable than hip and femur bones.

According to the texts and guidelines, in children with developmental disorders, this interpretation is more accurate based on skeletal growth with bone age assessment.^10^

It would be confusing for physicians if any of these points not to be considered and might lead to incorrect interpretation, diagnosis, and treatment in these patients. At the moment, these points are not considered in bone densitometry centres.

Other factors contributed to bone density disorders, including malnutrition, chronic infection, vitamin D deficiency, hypogonadism, delayed puberty, and decreased physical activity.^11^ This study aimed to evaluate bone density based on chronological age and compare it to bone age in chronic patients referred to the bone densitometry centre of the Children’s Medical Centre Hospital.

## METHODS

### Study design and participants

This prospective cross-sectional study has been done on 80 patients who referred to the bone densitometry centre of Children’ Medical Centre over a period of one year (July 2019 to July 2020). Along with DEXA method bone densitometry in patients, left wrist radiography was also performed for determination of bone age and to determine Z-score based on chronological and bone age.

### Inclusion and exclusion criteria

Patients with a history of oral or long-time injectable corticosteroid use, referred rheumatologic patients, referred nephrotic syndrome patients, post-transplantation patients, patients after cancer recovery, CF patients, major thalassemia patients, patients with gastrointestinal and malabsorption problems and all chronic patients who had an indication to screening studies, were studied based on screening protocol. Patients under treatment with bisphosphonates, patients with disorders like skeletal dysplasia, imperfect osteogenesis, chronic renal failure, and congenital adrenal hyperplasia were excluded from the study.

### Data collection and statistical analysis

A questionnaire containing demographic information, type of disease, duration of illness, drug history, history of fractures, bone pain, physical activity, and calcium and vitamin D intake, was completed for all patients. Also, serum level of calcium and vitamin D metabolites were checked for all patients if had not checked before. BMD of the lumbar spine (L2-4) using X-ray energy dose (DEXA) (Hologic QDR1000W / 892; Hologic, Boston, MA) was determined and expressed in grams per square centimetre. The low-density algorithm was used for values below 0.5 g / cm^2^. The coefficient variation for measuring repetition was 0.4% for 1 *gr* and 0.8% for 0.5 *gr*. The data obtained from this study were analysed with IBM SPSS version 22 software. Chi-Square tests were used to analyse the qualitative data between two groups and, if necessary, Fisher exact test was used. Paired-t test and, if necessary, Mann-Whitney U test were used to analyse quantitative data between two groups. Also, the statistical significance level was considered to be 0.05.

## RESULTS

A total of 80 patients who referred to the bone densitometry centre of Children’ Medical Centre from July 2019 to July 2020 were eligible for study. 42 of them (52.2%) were female and 38 patients (47.5%) were male. The mean weight of the patients was 36.12± 16.8 kg, the mean height was 137.94± 26.44 cm, the mean body mass index (BMI) was 18.52± 8.26 kg / m^2^, the mean age of them was 11.5±4.3 years (2.25–26.5) and their mean bone age was 10.27 ± 3.64 years. The mean Z-score of chronological age and bone age for spinal cord was −0.8±1.85 years and −0.58±1.64 years; respectively.

The mean Z-score of chronological age and bone age for the femur was −1.6 ± 1.02 and −1.32 ± 1.4 years; respectively. The difference between the Z-score of chronological age and bone age was not significant for spinal cord, despite, it is statistically significant for the femur. In this study, 12 patients underwent bone marrow transplantation. Serum level of Alkaline phosphatase was significantly higher in these patients compared to the other ones.

The mean Z-score of chronological age for spinal cord and Z-score of chronological age and bone age for the femur were significantly low compared to the other patients. In such cases taking corticosteroids, the difference of Z-score of the chronological age and bone age of spinal cord and femur was statistically significant. In patients who had a bone marrow transplantation, although the difference of the Z-score of chronological and bone age of the femur was significant but it was not significant for the spinal cord. In patients with cystic fibrosis (CF), the difference of the Z-score of bone age and chronologic age of spinal cord was significant, but it was not significant for the femur.

In patients with rheumatologic disorders, the difference of Z-score of the chronological age and bone age of spinal cord and the femur was significant. In patients with Hematologic disorders, the difference of Z score of the chronologic age and bone age of the femur was significant, but this difference was not significant for the spinal cord.

In these patients, the mean serum level of calcium was 10.12+10.43, the mean serum level of phosphorus was 3.56+ 0.976, the mean serum level of alkaline phosphatase was 608.4+ 262.97and the mean serum level of Vitamin D was 30.69+ 9.43. 58% of patients had Fanconi Anaemia and 4% of them had Scleroderma. Of all patients, 33 patients (41.3%) were in the first stage of maturity, 14 patients (17.5%) in the second stage of maturity, 14 patients (17.5%) in the third stage of maturity, and 14 patients (17.5%) in the fourth stage of maturity and 5 patients (6.3%) in the fifth stage of maturity.

Two patients had limited daily movements and three of them had no movement, but the remaining (75 cases) had normal physical activity. Of all patients, 23 patients (28.8%) did not take calcium and 24 patients (30%) did not take vitamin D. Of all patients, 56 patients (70%) had a history of prednisolone intake, and 14 patients (17.5%) had a history of concomitant use of prednisolone and methotrexate. Bone pain was seen in 20 patients (25%) and 12 patients had a history of bone fractures (15%). The mean of laboratory, demographic, and bone density variables were not significantly different between the group taking prednisolone and the group that did not take prednisolone (**Table 1**). The mean of laboratory and demographic variables showed a significant difference between the group that had physical activity and the group that did not. However, the mean Z-score of bone age and chronological age of the spinal cord and the Z-score of chronological age and bone age of the femur were significantly different between two groups (**Table 2**). The mean of BMI, serum calcium, phosphorus, alkaline phosphatase, Z score of chronologic age of spina cord, Z score of chronologic age, and bone age of femur were not significantly different between the group with normal puberty and the group with puberty failure, but, the mean of height, mean Z score of bone age, weight, vitamin D, and Z Score of bone age of the spinal cord were significantly different between these two groups (**Table 3**).

**Table 1.** Comparison by demographic, laboratory, and bone density by Prednisolone use.

**Variable**	**Prednisolone consumption**	**Number**	**Mean**	**Std. Deviation**	**P-value**
Bone age	No	22	10.2295	4.07748	0.956
	Yes	53	10.2811	3.47949	
Height	No	24	140.875	21.1296	0.519
	Yes	56	136.679	28.4912	
Weight	No	24	36.292	18.2982	0.954
	Yes	56	36.054	16.3311	
BMI	No	24	17.5629	5.48151	0.500
	Yes	56	18.9352	9.21473	
Ca	No	24	9.071	.6981	0.557
	Yes	56	10.579	12.4665	
P	No	23	3.552	.9999	0.632
	Yes	55	3.565	.9627	
ALP	No	23	612.087	297.6551	0.936
	Yes	50	606.700	248.6521	
Vit D	No	24	31.0000	7.70658	0.848
	Yes	54	30.5509	10.16272	
Spine bone age Z score	No	23	−.709	1.6197	0.667
	Yes	56	−.527	1.7350	
Chronological Spine age Z score	No	24	−.8417	1.64367	0.904
	Yes	56	−.7868	1.94735	
Femur bone age Z score	No	23	−1.0843	1.12185	0.237
	Yes	56	−1.4196	1.14279	
Chronological Femur age Z score	No	23	−1.596	1.0734	0.970
	Yes	56	−1.607	1.2656	

**Table 2.** Comparison of demographic, laboratory findings, and bone density of studied patients by presence of physical activity.

	**Physical activity**	**Number**	**Mean**	**Std. Deviation**	**P-value**
Bone age	No	70	10.4493	3.45634	0.326
	Yes	5	7.7000	5.45837	
Height	No	75	139.693	24.7091	0.190
	Yes	5	111.600	39.7844	
Weight	No	75	37.080	16.5217	0.476
	Yes	5	21.800	16.4073	
BMI	No	75	18.6949	8.48177	0.392
	Yes	5	15.9520	2.91460	
Ca	No	75	9.003	.7930	0.710
	Yes	5	26.980	41.9394	
P	No	73	3.582	.9684	0.848
	Yes	5	3.260	1.0040	
ALP	No	68	584.735	247.4132	0.059
	Yes	5	930.200	283.7432	
Vit D	No	73	31.0925	9.48328	0.490
	Yes	5	24.8000	6.76018	
Spine bone age Z score	No	74	−.446	1.5899	0.006
	Yes	5	−2.560	2.1431	
Chronological Spine age Z score	No	75	−.6581	1.76099	0.004
	Yes	5	−2.9800	2.00050	
Femur bone age Z score	No	74	−1.1992	1.03762	0.000
	Yes	5	−3.1400	1.13049	
Chronological Femur age Z score	No	74	−1.484	1.1241	0.000
	Yes	5	−3.380	1.0450	

**Table 3.** Comparison of demographic, laboratory findings, and bone density of studied patients by Normal Tanner puberty stage.

	**Normal Tanner puberty stage**	**Number**	**Mean**	**Std. Deviation**	**P-value**
Bone age	Yes	48	9.0604	3.59482	0.001
	No	27	12.4093	2.61343	
Height	Yes	52	132.462	28.0570	0.011
	No	28	148.107	19.8221	
Weight	Yes	52	32.404	14.2763	0.006
	No	28	43.036	19.1669	
BMI	Yes	52	16.9640	3.11795	0.081
	No	28	21.4196	12.96108	
Ca	Yes	52	10.912	12.9028	0.362
	No	28	8.668	.7424	
P	Yes	50	3.438	.8564	0.168
	No	28	3.782	1.1219	
ALP	Yes	48	589.354	244.7180	0.395
	No	25	644.960	296.7657	
Vit D	Yes	51	32.2696	9.51795	0.041
	No	27	27.7037	8.65054	
Spine bone age Z score	Yes	52	−.269	1.6749	0.023
	No	27	−1.178	1.5943	
Chronological Spine age Z score	Yes	52	−.5385	1.71603	0.181
	No	28	−1.2950	2.01989	
Femur bone age Z score	Yes	51	−1.2510	1.16985	0.458
	No	28	−1.4514	1.09198	
Chronological Femur age Z score	Yes	51	−1.447	1.1882	0.120
	No	28	−1.889	1.2069	

Among children with different underlying diseases, the mean Z score of bone age and serum level of calcium were significantly different, but the difference between other variables in the healthy children group and the children with different underlying disease group was not significant.

The mean of serum alkaline phosphatase, Z score of the chronologic age of spinal cord, Z score of chronologic age, and bone age of the femur were significantly different between the groups who underwent bone marrow transplantation and patients with other treatments, but the differences among the other variables were not significant (**Table 4**).

**Table 4.** Comparison of demographic, laboratory findings, and bone density of studied patients by Normal BMT.

	**BMT**	**N**	**Mean**	**Std. Deviation**	**P-value**
Bone age	No	64	10.3539	3.48504	0.617
	Yes	11	9.7545	4.58478	
Height	No	68	138.485	26.9447	0.662
	Yes	12	134.833	24.1805	
Weight	No	68	36.882	17.1791	0.341
	Yes	12	31.833	14.5779	
BMI	No	68	18.8949	8.82565	0.342
	Yes	12	16.4192	3.18450	
Ca	No	68	8.979	.8222	0.896
	Yes	12	8.975	.7213	
P	No	67	3.515	.9826	0.448
	Yes	11	3.845	.8537	
ALP	No	61	580.377	249.1680	0.039
	Yes	12	750.833	295.8436	
Vit D	No	66	31.3053	9.34868	0.177
	Yes	12	27.3000	9.53215	
Spine bone age Z score	No	67	−.431	1.6884	0.065
	Yes	12	−1.408	1.5365	
Chronological Spine age Z score	No	68	−.6332	1.83380	0.050
	Yes	12	−1.7667	1.71482	
Femur bone age Z score	No	67	−1.1827	1.05805	0.009
	Yes	12	−2.1000	1.31079	
Chronological Femur age Z score	No	67	−1.464	1.1066	0.014
	Yes	12	−2.383	1.4782	

The difference of the mean of the demographic, laboratory variables, and bone density, between two groups of patients with bone pain and other patients was not significant (**Table 5**).

**Table 5.** Comparison of demographic, laboratory findings, and bone density of studied patients by Fx History.

	**Fx History**	**N**	**Mean**	**Std. Deviation**	**P-value**
Bone age	No	63	10.1103	3.70000	0.399
	Yes	12	11.0833	3.30862	
Height	No	68	137.500	26.3894	0.727
	Yes	12	140.417	27.7405	
Weight	No	68	35.353	15.3319	0.487
	Yes	12	40.500	24.0284	
BMI	No	68	18.4718	8.70844	0.895
	Yes	12	18.8167	5.31330	
Ca	No	68	8.947	.8278	0.405
	Yes	12	9.158	.6501	
P	No	67	3.639	.9625	0.460
	Yes	11	3.091	.8983	
ALP	No	62	627.258	244.2271	0.147
	Yes	11	502.091	345.5956	
Vit D	No	66	31.4773	8.98176	0.083
	Yes	12	26.3542	11.00850	
Spine bone age Z score	No	67	−.591	1.7202	0.890
	Yes	12	−.517	1.6090	
Chronological Spine age Z score	No	68	−.7568	1.86112	0.595
	Yes	12	−1.0667	1.85145	
Femur bone age Z score	No	67	−1.2648	1.18931	0.890
	Yes	12	−1.6417	.77161	
Chronological Femur age Z score	No	67	−1.536	1.2341	0.294
	Yes	12	−1.983	.9953	

## DISCUSSION

The results of this study showed that the difference between Z-score of bone age and chronological age of femur in all chronic children was significant (mean Z-score of bone age and chronological age of femur and spine between patients with various underlying diseases (including cystic fibrosis, Rheumatologic, blood, immunological, neurological, endocrine, and nephritic syndrome disorders (no significant difference). There was also a significant difference between the Z age of bone age and the chronological age of the femur in patients with rheumatic diseases, blood disorders, patients with a history of corticosteroids and bone grafts. The difference between Z-score and bone age and chronological age of the spine was also statistically significant in patients with cystic fibrosis, patients with rheumatic disorder and patients with a history of corticosteroids.

Based on the results of this study, none of the variables were significantly different between the group with a history of prednisolone use and other patients. Also, children who did not have normal physical activity had significantly lower chronological and bone age Z-scores on both spine and femur than patients with normal physical activity. In patients who did not have normal puberty, vitamin D and Z-score levels had lower bone age in the spine than in other patients. Finally, patients who underwent bone marrow transplantation had higher alkaline phosphatase, lower chronological age of the spine, and lower chronological and skeletal age of the femur than other patients.

Dr. Tabatabai et al. conducted a study to evaluate the rate of bone density determination by DXA method in the population of 10–20 years old in Tehran. In this study, bone density showed a positive correlation with age, height, weight, and puberty. They found that calcium intake was correlated with femoral bone density, while this correlation was not observed in spinal cord.^12^ In 2005, a study by Pludowski et al. evaluated 151 healthy children aged 4–18 years and 61 children aged 5–20 years with bone disorders to compare the chronologic age and bone age obtained by DXA method and found a strong correlation between chronologic age and bone age in both genders. Finally, they concluded that the simultaneous use of chronological age and bone age provides valuable information about the status of bone maturity.^13^ In 2019, a study by Zougbi et al. investigated the relationship between chronologic age and bone age calculated by DXA method and they found that, Z-score of bone age was less than chronologic age and finally concluded that bone age along with the chronologic age, helps to evaluate the status of patients with Duchenne muscular dystrophy who are under treatment by corticosteroids.^14^ According to the findings of the present study, in general, the difference between chronological age and bone age was not significant among all patients for spinal cord, but it was significant for femur bone, which indicates that the Z score of the chronological age of femur can be used to assess the status of patients. The study also showed that in patients taking corticosteroids, the differences in chronological and bone age of the spinal cord and femur were statistically significant. Also, in patients who underwent bone marrow transplantation, the difference between the chronologic and bone age of the femur was significant, but this difference was not significant for spinal cord.

In 2004, a study by Rita Ujhelyi et al. conducted to investigate the bone mineral density and bone homeostasis in cystic fibrosis patients and the changes over a two-year period, which showed that the bone age in adolescents was lower than the chronologic age. The BMD Z score of the lumbar and femoral neck area in each age group was less than normal.^15^ In the present study, the mean Z-score of bone age of patients was 11 years and the mean Z score of bone age of spinal cord and femur was −1.534 and −1.7 years, respectively.

In 2014, Janneke Anink et al. studied on bone age in patients with juvenile arthritis which showed that chronic inflammation along with glucocorticoid therapy and sedentary treatment exposed idiopathic juvenile arthritis (JIA) patients to an increased risk of growth retardation and decreased BMD. Use of Glucocorticoids associated with delay in bone age and female sex with low a score.^8^ In 2019 a study by Marushko et al. evaluated the serum level of vitamin D and status of bone density in the adolescent with JIA and showed that serum level of vitamin D was low in 92% of patients and 60% of patients had a Z score below − 2 in bone densitometry (16). In the present study, the mean bone age of the spinal cord and femur was 0.013 and −1.05, respectively and the mean level of vitamin D in these patients was 32.65 ng/mL.

A study by Dr. Kosarian et al. conducted to investigate bone density and related factors in thalassemia major and intermedia patients. A cross-sectional study was performed on 125 patients with thalassemia major and intermedia who underwent blood transfusion in special centers in Sari-Iran and showed that according to the Z-score, 61% of the cases were osteopenic. The correlation of age with low BMD was significant. They concluded that bone density measurement should be routinely performed in these patients.^17^

In 2003, a study by Dr. Shamshirsaz et al. aimed to evaluate BMD using the DEXA method in 212 thalassemia patients aged 10 to 20 years living in Tehran and determine the potential risk factors. There was not any significant difference in the severe decrease in bone density of spinal and femoral bones between boys (spinal 46.4%, femur 11.2%) and girls (spinal 54.7% and femur 10.4%). Reduced bone density found at 39.5% of the spinal bones and 37.5% of the femur bones. Patients with severely decreased bone density in the spinal bones were significantly older (P <0.001). The data obtained from this study confirmed a significant decrease in bone density in Iranian thalassemic patients. Although more research is needed, well-known prophylactic methods such as hormone therapy and rapid treatment of bone density disorders have been emphasized in thalassemic patients.^18^ In this study, the mean bone age in patients with blood disorders (thalassemia major, Fanconi anaemic, acute lymphoblastic leukaemia [ALL] and acute myeloid leukaemia [AML]) was 10.88 years. The mean Z score of bone age of spinal cord and femur was −0.781 and −1.51; respectively. Serum levels of vitamin D and calcium were 8.9 and 28.6; respectively. In total, 12 patients underwent bone marrow transplantation in this study. Serum level of alkaline phosphatase was significantly higher in these patients than others, and the rate of bone age and the chronological age of the spinal cord and bone age of the femur were significantly lower than other patients. In 2007, Fatemeh Sayarifard et al. Studied on the bone density and diabetes in children and concluded that type 1 diabetes (DM) causes changes in BMD. The mean age was 12.5 years (4–14 years). Low BMD was detected in the lower normal range in 17 (15.2%) and 25 (22.3%) patients, respectively. There was a significant correlation between BMD and patient’s age and age of diabetes diagnosis, IGF-1, HbA1c, and PTH, but only an increase in HbA1c level effectively predicted a decrease in BMD.^19^ In 2011, J Feber et al. studied the effect of glucocorticoid in patients with nephrotic syndrome on bone age and bone density. They found that there was a reverse relationship between exposure to glucocorticoids and Z-score of spinal BMD and a small number of spinal lesions (8%).^20^ In the present study, 8 patients had nephrotic syndrome. The mean bone age in these patients was 10.9 years.

The mean of Z scores of chronological age and bone age of spinal cord were −1.74 and −0.4; respectively, and the mean of Z scores of chronological age and bone age of femur were −1.02 and −0.81; respectively.

According to some studies, there is no correlation between calcium intake in diet and bone mineral content in childhood.^21–22^ However, in a prospective study on twins, the results showed that daily intake of 1000 mg of calcium significantly improved BMD.^23^ Low bone density increases the risk of bone fractures and leads to increased mortality and morbidity in communities.^24^ Low bone density in an adult can indicate a lack of maximum bone density during the early years of life.^25–27^ Therefore, early diagnosis of at-risk individuals can reduce the risk of future bone fractures. During the years of growth, the amount of bone density is affected by the rate of skeletal system growing and increases with age. Bone minerals, which increase during the early years of life, decrease in the last years of life.^28^ If bone density reaches to its desired level during childhood and adolescence, it would have a key role in preventing osteoporosis in the future.^29^

## CONCLUSION

The results of the present study showed that in all patients, the difference between Z-score of chronological age and bone age was not significant for spinal bone, but statistically significant for femur. The results also showed that corticosteroid intake could cause a difference between Z-score of chronological and bone age of the spinal cord and femur, and bone marrow transplantation could cause a difference between the Z-score of chronological age and bone age of the femur but have no effect on spinal cord. It is recommended that in infants with chronic diseases especially in infants were under corticosteroid therapy, in addition to chronological age we should use z-score of bone age in larger scale and with a larger number of samples over a longer period of time.

## References

[1] Di Iorgi N Maruca K Patti G Mora S. Update on bone density measurements and their interpretation in children and adolescents. Best Pract Res Clin Endocrinol 2018;32(4):477–98. 10.1016/j.beem.2018.06.002 30086870

[2] Ezaddini–arkani F Navvab-azam A Bahshardoost N Mansourian H Ahmadieh MH Sadat-Hoseini SA. Investigating the correlation between calendar age, skeletal age, dental age and estimated age of panoramic radiography in patients referring to Yazd Dental Clinics in 2004–2005. J Dent Sch Shahid Beheshti Univ Med Sci 2006;24:474–84.

[3] Reinhardt RA Payne JB Maze CA Patil KD Gallagher SJ Mattson JS. Influence of estrogen and osteopenia/osteoporosis on clinical periodontitis in postmenopausal women. J Periodontol 1999;70(8):823–8. 10476887 10.1902/jop.1999.70.8.823

[4] Leite HR O’Reilly MT Close JM. Skeletal age assessment using the first, second, and third fingers of the hand. Am J Orthod Dentofacial Orthop 1987;92(6):492–8. 3479895 10.1016/0889-5406(87)90231-9

[5] Płudowski P Lebiedowski M Lorenc RS. Evaluation of the possibility to assess bone age on the basis of DXA derived hand scans—preliminary results. Osteoporos Int 2004;15(4):317–22. 14615883 10.1007/s00198-003-1545-6

[6] Mughal AM Hassan N Ahmed A. Bone age assessment methods: A critical review. Pak J Med Sci 2014;30(1):211. 24639863 10.12669/pjms.301.4295 PMC3955574

[7] Müller L-SO Offiah A Adamsbaum C Barber I Di Paolo PL Humphries P Bone age for chronological age determination—statement of the European Society of Paediatric Radiology musculoskeletal task force group. Pediatr Radiol 2019:1–4. 30911781 10.1007/s00247-019-04379-4

[8] Anink J Nusman CM van Suijlekom-Smit LW van Rijn RR Maas M van Rossum MA. Automated determination of bone age and bone mineral density in patients with juvenile idiopathic arthritis: a feasibility study. Arthritis Res Ther 2014;16(4):424. 25158602 10.1186/s13075-014-0424-1 PMC4293113

[9] Bachrach L. Dual energy X-ray absorptiometry (DEXA) measurements of bone density and body composition: promise and pitfalls. J Pediatr Endocrinol Metab JPEM 2000;13:983–8. 11086651

[10] Morris EB Shelso J Smeltzer MP Thomas NA Karimova EJ Li C-S The use of bone age for bone mineral density interpretation in a cohort of pediatric brain tumor patients. Pediatr Radiol 2008;38(12):1285. 18769909 10.1007/s00247-008-0991-x PMC2693364

[11] Conway S Morton A Oldroyd B Truscott J White H Smith A Osteoporosis and osteopenia in adults and adolescents with cystic fibrosis: prevalence and associated factors. Thorax 2000;55(9):798–804. 10950902 10.1136/thorax.55.9.798 PMC1745849

[12] Tabatabaei S Shamshirsaz A Bekheinia M Larijani B Moradi lake M Tabatabaei M. Normative value of bone mineral density in 10–20 year-old Iranian adolescents. SJKU 2005;10(3):61–72.

[13] Płudowski P Lebiedowski M Lorenc RS. Evaluation of practical use of bone age assessments based on DXA-derived hand scans in diagnosis of skeletal status in healthy and diseased children. J Clin Densitom 2005;8(1):48–56. 15722587 10.1385/jcd:8:1:048

[14] Al-Zougbi A Mathews KD Shibli-Rahhal A. Use of bone age for evaluating bone density in patients with Duchenne muscular dystrophy: A preliminary report. Muscle Nerve 2019;59(4):422–5. 30636004 10.1002/mus.26413 PMC6417931

[15] Ujhelyi R Treszl A Vásárhelyi B Holics K Tóth M Arató A Bone mineral density and bone acquisition in children and young adults with cystic fibrosis: a follow-up study. J Pediatr Gastroenterol Nutr 2004;38(4):401–6. 15085018 10.1097/00005176-200404000-00007

[16] Marushko T Holubovska YY. Vitamin D status and bone mineral density in patients with juvenile rheumatoid arthritis. Childs Health. 2019;14(1):13–18.

[17] Kosaryan M Vahidshahi K Emadi Jamali A Sarparast L. Bone mineral density of patients with beta thalassemia, Thalassemia Research Center, 2007. J Mazandaran Univ Med Sci 2012; 21(86):63–73.

[18] Shamshirsaz AR Bekheirnia MR Kamgar M Tabatabaie SM Moradi Zirkuhi A Bouzari N Therapeutic indices in thalassemia major: association with bone mineral density. Payesh 2004;3(1):75–81.

[19] Sayarifard F Safarirad M Rabbani A Sayarifard A Ziaee V Status of Bone Mineral Density in Children with Type 1 Diabetes Mellitus and Its Related Factors. Iran J Pediatr 2017;27(4):e9062. doi: 10.5812/ijp.9062.

[20] Feber J Gaboury I Ni A Alos N Arora S Bell L Skeletal findings in children recently initiating glucocorticoids for the treatment of nephrotic syndrome. Osteoporos Int 2012;23(2):751–60. 21494860 10.1007/s00198-011-1621-2 PMC4000256

[21] Matkovic V Fontana D Tominac C Goel P Chesnut C 3rd. Factors that influence peak bone mass formation: a study of calcium balance and the inheritance of bone mass in adolescent females. Am J Clin Nutr 1990;52(5):878–88. 2239765 10.1093/ajcn/52.5.878

[22] Chan GM. Dietary calcium and bone mineral status of children and adolescents. Am J Dis Child 1991;145(6):631–4. 2035492 10.1001/archpedi.1991.02160060049019

[23] Johnston CC Jr Miller JZ Slemenda CW Reister TK Hui S Christian JC Calcium supplementation and increases in bone mineral density in children. NEJM 1992;327(2):82–7. 1603140 10.1056/NEJM199207093270204

[24] Johansson A Burman P Westermark K Ljunghall S. The bone mineral density in acquired growth hormone deficiency correlates with circulating levels of insulin-like growth factor I. J Int Med 1992;232(5):447–52. 10.1111/j.1365-2796.1992.tb00613.x 1453131

[25] Dawson-Hughes B Harris SS Krall EA Dallal GE. Effect of calcium and vitamin D supplementation on bone density in men and women 65 years of age or older. NEJM 1997;337(10):670–6. 9278463 10.1056/NEJM199709043371003

[26] Mitlak BH Nussbaum SR. Diagnosis and treatment of osteoporosis. Annu Rev Med 1993;44(1):265–77. 8476248 10.1146/annurev.me.44.020193.001405

[27] Recker RR Davies KM Hinders SM Heaney RP Stegman MR Kimmel DB. Bone gain in young adult women. JAMA 1992;268(17):2403–8. 1404797

[28] Thomas KA Cook SD Bennett JT Whitecloud T 3rd Rice JC. Femoral neck and lumbar spine bone mineral densities in a normal population 3–20 years of age. J Pediatr Orthop 1991;11(1):48–58. 1988478 10.1097/01241398-199101000-00011

[29] Gimeno JB Azcona CSJ Sierrasesúmaga LA. Bone mineral density determination by osteosonography in healthy children and adolescents: normal values. An Esp Pediatr 2001;54(6):540–6. 11412400

